# 1180. Empiric treatment of uncomplicated intraabdominal infection with cefazolin with or without metronidazole

**DOI:** 10.1093/ofid/ofad500.1020

**Published:** 2023-11-27

**Authors:** Jaime Borkowski, Radhika S Polisetty, Rishita Shah, Jay Liu, Luis Manrique, Stephen Grohmann, Nicholas Johnson

**Affiliations:** NM Delnor Hospital, Geneva, Illinois; Midwestern University College of Pharmacy/ Northwestern Medicine Central DuPage Hospital, Winfield, Illinois; Northwestern Lake Forest Hospital, Lake Forest, IL, Illinois; Northwestern Medicine, Geneva, Illinois; Northwestern Medicine, Geneva, Illinois; Northwestern Lake Forest Hospital (Lake Forest, IL), Chicago, Illinois; Northwestern University, Pingree Grove, Illinois

## Abstract

**Background:**

Treatment guidelines for management of intra-abdominal infection (IAI) differ on whether cefazolin should be used first line to treat uncomplicated infection. In an effort to reduce unnecessary fluoroquinolone and piperacillin/tazobactam use, protocols were implemented with which recommendations were made to narrow to cefazolin ± metronidazole for uncomplicated IAI. The purpose of this study was to determine if there is a difference in outcomes with cefazolin +/- metronidazole versus broader-spectrum antibiotics for treatment of uncomplicated IAI.

**Methods:**

This was a retrospective cohort study that reviewed inpatients at three acute-care community teaching hospitals from January 1, 2017, through December 31, 2021. Treatment groups were patients treated with cefazolin +/- metronidazole (study group) compared to those treated with piperacillin/tazobactam or ceftriaxone, ciprofloxacin, or cefepime each +/- metronidazole (control group). Inclusion criteria were age ≥ 18 years old; admission diagnosis of uncomplicated cholecystitis, diverticulitis, or appendicitis; and received as empiric inpatient treatment either cefazolin ± metronidazole, piperacillin/tazobactam, or ceftriaxone, ciprofloxacin, or cefepime ± metronidazole for at least 24 hours. Exclusion criteria were presence of perforation or abscess for cholecystitis or diverticulitis, cholangitis or pregnancy. The primary outcome measure was all-cause 30-day readmission rate.

**Results:**

A total of 779 patients were included in the cefazolin group and 2269 patients were included in the broader-spectrum group. All-cause 30-day readmission rate was 5.8% in the cefazolin +/- metronidazole compared to 12.1% in the broader-spectrum group (OR 0.38, 95% CI 0.27-0.53, p < 0.001). Average length of stay was 3.6 days for the cefazolin group vs. 4.4 days for the broader-spectrum group (cefazolin group range 1 to 35 days, broader-spectrum group range 1 to 59 days, p < 0.0001).

**Conclusion:**

Empiric treatment of uncomplicated intra-abdominal infection with cefazolin +/- metronidazole did not result in an increase in 30-day readmissions compared to broader-spectrum empiric antibiotics.

**Disclosures:**

**All Authors**: No reported disclosures

